# Supplementary material for: Contributions to a neurophysiology of meaning: the interpretation of written messages could be an automatic stimulus-reaction mechanism before becoming conscious processing of information
Source: PeerJ. 2015 Oct 27;3:e1361. doi: 10.7717/peerj.1361 (PMC4627920; doi:10.7717/peerj.1361)
Supplement: Supplemental Information 1 — The Supplemental Materials file contains details, unsuitable to be presented in the manuscript, about the following subjects: Foundations of the chosen methodological approach— Technical documentation (research guidelines and protocol, the employed questionnaire and so on)—Analysis of the collected data suitability—Added materials aimed to a further check of our hypothesis. [file peerj-03-1361-s001.pdf]

Contributions to a neurophysiology of meaning: The interpretation of written messages could be an automatic stimulus-reaction mechanism before becoming conscious processing of information.

Roberto Maffei[*1], Livia Selene Convertini[1], Sabrina Quatraro[1], Stefania Ressa[1], Annalisa Velasco[1]

[1] A.L.B.E.R.T. (ARPA-Firenze Landmarks on human Behaviour Experimental Research Team), Florence – Italy.

* E-mail: roberto@robertomaffei.it ; albert@arpafirenze.it

SUPPORTING INFORMATION

Supporting Information

Summary

SECTION 0 – *About method*

Part I - Materials and Method

SECTION 1 – *The research guide-lines*

SECTION 2 – *The case: description and research's rationale*

SECTION 3 – *The research protocol*

SECTION 4 – *The questionnaire: message texts and questions (English translation)*

SECTION 5 – *Case structure and communication critical points*

Part II - The collected data

SECTION 6 – *About the sample*

SECTION 7 – *The harvest*

SECTION 8 – *Data quality check: compliance with research requirements and technical-theoretical questions related to answer interpretation*

SECTION 9 – *Data quality check: analysis of the collected data distribution*

Part III - Added materials

SECTION 10 – *Analysis of some indicated component distributions*

SECTION 11 – *Complement materials on coherence investigation*

SECTION 12 – *The "block preference" analysis*

References

Figures : Fig. S1 ; Fig. S2 ; Fig. S3 ; Fig. S4 ; Fig. S5 ; Fig. S6 ; Fig. S7 ; Fig. S8 ; Fig. S9 ; Fig S10 ; Fig. S11

Tables : Tab. S1 ; Tab. S2 ; Tab. S3 ; Tab. S4 ; Tab. S5 ; Tab. S6 ; Tab. S7 ; Tab. S8 ; Tab. S9 ; Tab. S10 ; Tab. S11 ; Tab. S12 ; Tab. S13

SECTION 0 – ***About method***

The naturalistic approach we chose presents several difficulties, given that human communication cannot actually be observed "from outside": it is part of us and we simultaneously belong to it; it is impossible to avoid interactions (i.e. "interference") with the studied sample. However, for research purposes, there is an at least partial solution: an external point of view can be simulated. We designed such simulation considering that a total exclusion of personal/relational factors is illusory, even with unknown persons, given that it is impossible to take under control their emotional involvement (their subjective reactions to the survey in itself and to survey conductors, independently of any specific content). On this basis, we made three operative decisions: on the specific subject in which to involve the sample; on the materials to be employed for data collection; on the survey modalities.

About the subject, we involved the sample in a real world-like communication case, neutral with respect to possible personal critical issues and totally external to the sample members' lives and to their possible relationships with the survey conductors. About the materials, we employed the complete sequence of the messages exchanged in the case, full-text versions, submitting them to the sample through a specifically designed questionnaire that alternated messages and questions in a precise sequence. About the survey modalities, we tried to transform the relational weak point in a strong one: we concluded that, in the end, the most effective condition could never be the illusory neutrality; rather, it could be the possibility to act in a stress-free condition, to read messages without time pressure, to let sensations and emotions emerge and to report

them without any fear. It is worth delving a little further in how we carried this last decision out: a friendly, familiar environment, with a known conductor (to reduce the structural initial difficulties in human relationships); a shared programming of the survey date and hour (to get the maximum possible of comfort and relax); the possibility to answer free from any constrictions (for this, we mainly used questions with opened answers); the certainty about anonymity and the non-evaluative purposes of the survey. At the same time, the consciousness of participating to a serious work and the guarantee (for the research's purposes) of mostly uniform survey modalities.

Two last considerations. The first is that we define our approach as “naturalistic” in that it is aimed to explore the interpretation process in the “natural” conditions in which it is performed: for example, in their communications, human beings usually deal with (and interpret) full messages; however, current laboratory approaches are forced to privilege the use of single words or isolated short phrases because of the difficulties associated to the study of free natural language interactions. We decided to check, through our “naturalistic” approach, the possibility to complement current studies and bring something new to scientific knowledge.

The second consideration regards the kind of control that, through our approach, we exerted on the survey: besides the rigorously standardized data collection procedure (see this Supporting Information, Section 1 and Section 3), our control mainly lied on the reliability and the homogeneity of the relational system, rather than on the (impossible) attempt to cut off the relational aspects.

PART I - Materials and Method

SECTION 1 – ***The research guide-lines***

Object to be investigated: human communication, the process through which a receiver attributes meaning to a message (i.e. the interpretation process, the way he/she "understands" the incoming message).

Methodological approach: given that research on human communication (H.c. from now on) has provided, about interpretation, abundance of theoretical hypotheses along with still indefinite answers, it seems a good solution to re-start from a basic exploration, which means from the **phenomenology** of specific events in a given environment ("naturalistic" approach).

Action plan: (1) Submitting a real world-like case to the sample and requesting the solution of a concrete problem related to it; (2) Observing respondents' reactions through collecting their accounts; (3) Analysing the respondents' accounts. The case should be suitable to be fully documented for the sample and its investigation should require a reasonably short time.

On the basis of these premises, the GUIDE-LINES for our investigation are established as it follows:

- The research will be carried out through a qualitative and quantitative statistics-based research.
- The sample will be randomly composed by adult Italians, granted with High-school degree (or upper education levels) and regardless of their student or employed (any employment) condition.

- About education level, possible exceptions only for people whose literacy, joined with their life experience, allow them to understand without effort the case documentation[1].
- The sample will be engaged in an appropriately documented H.c. case and individual reactions to it will be investigated through a questionnaire. The questionnaire will end posing a **concrete problem**, referred to the case, and requiring the respondent's solution.
- The case must be **quasi-real**, not a mere laboratory exercise. So, it will be based on real world cases, remaining as close as possible to reality, at the same time avoiding any reference or hint to the original real situations. It will be a written communication case (to allow for a better control on the stimuli submitted to the participants), short enough to be taken into account complete, unabridged and accomplished.
- The sample will collect about 100 individuals and the survey sessions must not exceed the 30 – 45 minute time range. The sessions may be attended individually or in groups, but the filling of the questionnaires will always be an individual act.
- All the survey sessions will take place under the control of a conductor, who will follow a standard procedure for presenting the texts about the case and the questions (in order to send homogeneous inputs to the sample).

[1] Actually, only 4 participants, out of the 102 composing the sample, had qualifications inferior than a High-school degree.

SECTION 2 – ***The case: description and research's rationale***

Introduction and rationale of the research. We examined, for our research, a series of real-world cases of interaction some of the authors had dealt with in their professional experience. The chosen cases were short enough to be easily handled and, at the same time, they were fully representative of the real world's complexity. The case to be created should have consisted of a realistic problem to challenge participants with; moreover, it should have been fully documented from start to end, consisting of written messages (e-mails) only and set inside an Italian corporation. We set up our case, we named it "The employee and the architect" (as a tribute to the protagonist characters) and we drew up the research protocol (see this Supporting Information, Section 3).

A complete description of the case can be found ahead in this present Section. In extreme synthesis, it goes on as an exchange of written messages (5 e-mails in total) between the employee and the architect; we have submitted these messages to the sample leading its members in a two-step work. In the first step, we have asked the participants to carefully read Messages #1, #2 and #3 in sequence; then, to interpret them and the situation they outline; finally, to report and display the "concrete elements" on which their interpretations were based. The rationale was: *in vivo* observation of the interpretation process, quali-quantitative analysis and formulation of a hypothesis.

In the second step, we have submitted to participants the last two messages asking them to read carefully the texts, to interpret them, then to solve a problem: the last two messages were two different versions of Message #4 and the problem was to indicate which of the two could have produced the final answer (fifth message). The rationale

was: exploring the relationship between interpretation and action and, through a quantitative analysis, obtaining a first check of our hypothesis.

Case details. What follows is a complete description of the case used for our research, from its start to its end.

TITLE: We named the case “The employee and the architect”, as a tribute to its protagonist characters.

CHARACTERS:

- XX – The employee. Female, line worker in an office of an Italian corporation. Her office is undergoing works regarding the heating plant.
- YY – The architect. Male, executive in charge of the works. He is a colleague of XX, being himself an employee of the corporation. He has superior position and duties, with respect to her, but he belongs to another branch and has no hierarchic power on her.
- Dr. KK and Dr. ZZ – Employee’s (XX) colleagues, just mentioned by the architect in reference to the works in progress.
- The Colleague – A shadow character in the interaction, as he never appears during the action. The architect (YY) requests his advice about the text of one message to be sent to XX.

NOTE: The employee and the architect do not know each other; this interaction is their first contact, started and ended through e-mails only.

The STORY:

Notice – The texts of the messages that will be mentioned here below can be found in this Supporting Information, Section 4. The first three messages are presented under the form of a description in order to make the whole situation more comprehensible to the reader of this Supporting Information; however, they have been submitted to the sample as full-text documents.

Prologue – Work on the heating plant is coming to its end; XX (the employee) starts the interaction by writing to the architect (Message #1). She requests an inspection for quality control on the basis of generically claimed issues.

YY (the architect) replies immediately (Message #2) declaring, very briefly and generically as well, that the situation has already been checked and lies under control.

Several weeks later, XX writes again (Msg #3) insisting for an inspection and indicating some specific issues at the basis of her claim. The tone of her message appears to be hardened and one passage seems to contain a sort of threat.

Action – YY prepares a new reply (Msg #4, “Hard” version, in short Msg #4/H) but requests his colleague an advice, before sending it. The colleague accepts YY’s request and suggests sending a different version (Msg #4, “Softer” version, in short Msg #4/S).

The architect accepts the advice, the “Softer” Msg #4/S is sent and the case ends with a last reply of XX (Msg #5) declaring her satisfaction.

NOTES: Because of a specific choice of YY’s colleague, Msg #4/S (the “Softer” version) bears the same content of the "Hard" (H) version but is written in different form

and its topics are put in a different sequence. Although XX expresses her satisfaction, no inspection has been carried out nor it has been requested any more.

SECTION 3 – ***The research protocol***

Notice – The texts of the messages that will be mentioned here below can be found in this Supporting Information, Section 4. The first three messages are presented under the form of a description in order to make the whole situation more comprehensible to the reader of this Supporting Information; however, they have been submitted to the sample as full-text documents.

The protocol:

INTRODUCTION

1. A case managed completely via e-mail, between an employee and a professional (the "architect"), has been set up. It concerns an interaction, related to a problem, inside an Italian corporation; the interaction lasts one month and a half. The problem developed and was completely solved through 5 transactions (5 messages were exchanged, chronologically labelled from #1 to #5). The employee starts the first transaction (Message #1) and concludes the interaction with the fifth one (Message #5).
2. During the action, the architect requests the opinion of a colleague of his; such request refers to a draft of the answer to Msg #3 spontaneously prepared by the architect (such draft is the first version of Msg #4, the "Hard"/H version). The colleague studies the case and proposes an alternative Msg #4 (the "Softer"/S version); the advice is accepted by the architect, the "S" version is sent and produces the expected result, as the last reaction of the employee demonstrates (Msg #5).

3. The used case is based on real cases which some of the authors had dealt with; it remains as close as possible to reality at the same time avoiding any reference or hint to the original real situations.

The QUESTIONNAIRE and its MANAGEMENT

4. Anonymity of respondents will be fully guaranteed during either the survey (questionnaire collection) or the analysis (data elaboration). No personal data will be asked; information that is necessary for statistical purposes (age, gender, education level and employment) will be requested as aggregated through pre-defined bins only.
5. For a better representation in the questionnaire, the case has been divided into two parts. In the first part (corresponding to the “Prologue” of the case description, see this Supporting Information, Section 2), the first 3 messages are gathered, in the same order they have been sent. These messages have been printed in sequence, in a single A4 page. The aim of this first part is to collect data about the interpretation process in general through a first set of questions. Such questions have been printed in another single A4 page (two opened questions, #1 and #2, the first sub-divided into three sub-questions).
6. In the second part (corresponding to the “Action” of the case description, see this SI, Section 2), the two versions of Msg #4 (the "Hard" and the "Softer" one) are presented, in separate A4 pages. They are submitted to participants in sequence (not simultaneously) and the remaining questions are printed in a last A4 page. At first (Questions #3 and #4) the participants’ opinions are

requested (separately) about the presumable effects of each version of Msg #4 on XX. In the end, after the transcription of the very brief Msg #5 (the employee's last reply), participants are requested (Final Question) to indicate which version (the "Hard" or the "Softer"), in their opinion, has produced the effect showed in Message #5. The aim of this second part is to collect data about the relationship between the interpretations of the alternative messages and the action (the choice) performed by participants.

7. All the questions (or sub-questions, if present) have been divided into two parts: in the first one, the interpretation of the respondent about one specific aspect is requested. In the second one, he/she is invited to "indicate the concrete elements (words, sentences, expressions etc…) on which your answer is based".
8. A special attention has been dedicated to the wording of the questions. Structural ambiguity of natural language implies the impossibility to formulate sentences that can be univocally interpreted by everybody, as the acknowledged Italian linguist De Mauro confirms[2]. Thus, any idea to pursue completely unambiguous formulations has been dropped. After the first careful formulation of the questions, two pilot-sessions will be set up for testing the questionnaire's suitability and gather indications about possible

[2] The author (De Mauro, 1980) says that natural language is "equivocal" in etymological sense, from Latin *aeque vocare* (to name in the same way). That is: a same word can be used to refer to different things; different words can be used to indicate the same thing.

corrections. In addition, ex-post specific controls will discard from quantitative analysis all the possibly remained ambiguous cases.

9. Same attention has been dedicated to possible statistical distortion effects. For example the YY's Colleague opinion on Msg #4/H (the original, "Hard" version) could influence respondents inducing some biases in their final choice; furthermore, there could be a possible precedence effect if the two versions of Msg #4 were submitted always in the same order. On these bases, the presentation of the two versions to the participants will be counterbalanced: all the participants will be informed that they are going to see, at first, the version spontaneously prepared by the "architect". The second (the "alternative" version) will be presented as suggested to him by one of his colleagues when asked for an advice. However, about one half of the sample will actually receive the two versions in that order (first Msg #4/H, then Msg #4/S); the remainder will receive them in the reverse order.

SURVEY and DATA COLLECTION:

10. All the conductors of the survey sessions (12 persons, in total) are members of the research group or in contact with it. Non-members will follow a brief training, led by one of the authors. All the conductors are committed to avoid expressing any comment about the message texts and concentrate on survey process conformity. Conductors have also to assure that the process is clear for the participants and that they understand the structure of the case and the questions. In order to minimize the speech necessities for the conductors, a

title page has been prepared; it contains a presentation of the survey and the main context information (see this SI, Section 4). The conductors are due to invite participants to carefully read it. In the title page, the case will be presented as a real world case.

11. Informed consent will be requested verbally, after the reading of the title page. Written consent will not be collected for two reasons: the first is that it would imply the creation and management of a general database, paradoxically increasing, by its mere existence, the risks of accidental data diffusion. The second reason is that our data collection procedure (see also following points) anyway fully guarantees anonymity of participants. At the end of data collection, it will be impossible for everyone either to trace back participants starting from the filled questionnaires or to reconstruct the participants' list.
12. The 12 conductors will operate in a completely independent way and the participants will be enlisted by using their personal relationship network, extended until the third degree of separation. Enlisting requirements: adult condition (age>18 years), High-school degree at least. Exceptions about education level are accepted just for people whose literacy and life experience allow them to understand the case documentation without effort (see Note 1).
13. The conductors will collect questionnaires bereft of every personal indications (or even hints) related to participants. They will individually deliver the collected anonymous questionnaires to the authors' team and those documents will be randomly numbered and stored in a dedicated collection box. The

research activities that will follow (data entry, in order to set up a digital data base, and qualitative and quantitative analysis) will be performed on such anonymous database.

NOTE: Once the protocol defined, two successive pilot sessions have been set up (7 and 5 people respectively) and these experiences helped to progressively refine the form of the questions, until the definitive shape was reached. The texts of the messages remained always unaltered. The following Section 4 presents the questions in their final form.

SECTION 4 – *The questionnaire: message texts and questions (English translation)*

**Questionnaire summarizing form**			
Part / Question #	***n. of sub-quest.***	***n. of items***	***NOTES***
**Title page**	---	---	Presentation of the research and general instructions to participants
**Statistical information**	---	---	Gender, age range, education level, employment
**Question #1**	3	**2 x 3 = 6**	Opened answers
**Question #2**	**2**	**1**	Closed answer
		**2**	Opened answers
**Question #3**	---	**2**	Opened answers
**Question #4**	---	**2**	Opened answers
**Final Question**	---	**1**	Closed answer
		**1**	Opened answer
Total of 5 questions	*Total of 8 quest. / sub-questions*	*Total of 15 items*	*Total of 2 closed answers and 13 opened answers*

Title page

First of all, welcome and thank you for joining our research.

The e-mails on which this study is based will be submitted to you during the present session. They have been exchanged in a real working environment and they refer to an interaction that occurred in real life. They are presented in their original version; their text has not been modified to be used for this research. Of course, all the elements that specifically refer to persons, or to the real context, have been removed or appropriately altered for privacy reasons.

Your task consists in reading the messages, respecting their submission sequence. Please, read carefully and answer the questions intuitively, not analytically (although, not excluding some personal reflections, if necessary). Underline the text, take notes or look back at the message text, when deemed necessary, any time you need it.

All the questionnaires will be anonymous. We only ask you to give us general information about yourself, here below, for merely statistical purposes (data disaggregation).

[*Questions followed on gender, age range, education level and employment (answers requested through pre-defined bins only).*]

Message #1 (*description*)

A female line-worker (the "employee", named "XX") writes a 67 word e-mail to the Project Account (the "architect") about the installation of the heating plant in her office. She requires an inspection, claiming about "flaws" in the present state of the works. Flaws are no better detailed. In her request, she declares that she is also speaking in the name of some colleagues and she uses the expression: "we would be pleased if, at least once, someone of our Corporation would come here and control…".

Message #2 (*description*)

The Project Account (a male professional, the "architect", named "YY") answers to XX. In his brief message (48 words) he declares regularity in the Project progress, and ends with: "at the moment, the progress substantially complies with the chronogram".

Message #3 (*description*)

XX replies to YY's answer declaring herself totally unsatisfied. Her message (136 words) presents two main features: (i) some minor flaws are listed; (ii) she expresses what resembles an actual threat against YY, in the case he would not take measures regarding to the presented problem (she specifically refers to a hypothetic "waste of public money", given that the Project funding involved public sources).

QUESTIONS #1 and #2, about Messages #1, #2, #3 (*full text*)

1 * Please, read Messages #1 and #2 and answer to the following questions:

a - What do you think is going on, between XX and YY?

Could you indicate the concrete elements (words, sentences, expressions etc…) on which your answer is based?

b - In particular, how would you define XX's position during the interaction?

Could you indicate the concrete elements (words, sentences, expressions etc…) on which your answer is based?

c – How would you define, then, YY's position during the interaction?

Could you indicate the concrete elements (words, sentences, expressions etc…) on which your answer is based?

2 * Please, read Message #3 and answer to the following questions:

Do you think the attitude of XX towards YY has changed, in respect to Message #1?

[*YES/NO*]

If it has, how would you define the new XX's position, in respect to YY?

Could you indicate the concrete elements (words, sentences, expressions etc…) on which your answer is based?

Message #4 / "H" version (*the spontaneous "Hard" version by the architect, full text*)

Block #1

From: YY (*Project Account for the heating plant works*)
To: XX (*Employee in one of the offices affected by the works*)
Cc: ZZ (*Office referent for the works*)
Sent: … [*date*] [*hour*]
Subject: R: heating plant

Dear Mrs. XX,

Block #2

I want to premise that, for the sake of a wise management of the work process, intended to optimize the utilization of our Corporation resources (exactly, in order to avoid wasting public money):

- Before Project start, I asked the Director of your structure (B wing of the building), Dr. KK, to put a specific person in charge of controlling the work's progress;
- As far as I am concerned, the indicated person is, and will remain, Dr. ZZ;
- Dr. ZZ carefully planned the project development steps with us;
- Each office, situated in the B wing of the building, has been already supplied with heating systems (hardware), fully complying with the timetable agreed with Mrs. ZZ;
- The heating plant is now working, even though in provisional mode.

I do recommend you to send any communication, concerning the mentioned Project, to the specific person in charge of controlling, in order to avoid (as already happened) message exchange with personnel that is not directly and formally involved within the process.

Block #3

However, I inform you that, at the moment, the works under discussion have been suspended, in order to enable the provisioning of the plant-control software. It will manage automatically the heating system in the offices, including yours, regulating the warm air diffusion (in order, as said above, to reduce any waste of money).

As soon as the software will be installed by the contractor, the works will come to end. By the way, in this phase they should not affect the rooms situated in the B wing of the building at all, but only the thermo-station.

All quantitative and qualitative controls, requested by the CHK form [*formal inspection document*], will be carried out after the end of the works and just before their compliance to fixed quality standards will be attested, as prescribed by the current rules.

Block #4

This said, I have found your objections very interesting. For this reason, once the real existence of the problems you have marked will be assessed, I will certainly solve them as a part of my duty.

<u>*Block #5*</u>

Yours sincerely

The Project Account

Arch. YY - [Corporation branch] …………………

Message #4 / "S" version (*the "Softer" version suggested by YY's colleague, full text*)

<u>*Block #1*</u>

From: YY (*Project Account for heating plant works*)

To: XX (*Employee in one of the offices affected by the works*)

Cc: ZZ (*Office referent for the works*)

Sent: … [*date*] [*hour*]

Subject: R: heating plant

Dear Mrs. XX,

<u>*Block #2*</u>

I remember your last message, which I have already answered, and now I really thank you for this new one. In fact, we do believe that the attention of our colleagues, on field operating with structures and plants we provide, is fundamental to complete our tasks at best.

<u>*Block #3*</u>

In order to optimize our contribution, I have been since the beginning asking for a unique person in charge of controlling the works, accounted for your office's building. This person is Doctor ZZ (I might have already mentioned her in my previous answer even though, at present time, I am not certain about this). Her duty is to collect all the observations expressed by the staff about the work in progress, then to send it directly to my office. I think you already know her and she is going to receive a copy of the present message. I thought this would make communication easier.

<u>*Block #4*</u>

Concerning your request, you can be certain that, so far, our Project has been developed by following all the technical and formal standards prescribed by the current rules. In addition, I inform you that the works are not yet concluded and final checks (along with possible inspections) are about to be carefully planned. Please, inform your colleagues about the existence of a person in charge of control and do not hesitate to contact her in the case of further observations or possible problems. As I said, she will return your indications to us; this way, I assure you they will not be ignored.

<u>*Block #5*</u>

Best regards

The Project Account

Arch. YY - [Corporation branch] …………………

QUESTIONS #3 and #4, about Messages #4/H and #4/S (*full text*)

***Premise**: YY prepares Message #4 as an answer to Message #3 (received from XX). Before he sends it, he consults one of his colleagues, who advises him against sending and suggests a different text (**alternative** Message #4).*

3 * Please, read Message #4 and answer to the following questions:

<u>In your opinion, what effect will this version produce on XX</u>?

Could you indicate the concrete elements (words, sentences, expressions etc…) on which your answer is based?

4 * Please, read* alternative *Message #4 and answer to the following questions:

<u>In your opinion, what effect will the alternative version produce on XX</u>?

Could you indicate the concrete elements (words, sentences, expressions etc…) on which your answer is based?

Message #5 (*full text*)

Thank you very much for your interest and for the information. That was very kind of you and your answer was exhaustive.

Best regards

XX

FINAL QUESTION

Consider that Message #5 was the final reaction of XX and answer the following questions:

<u>In your opinion, which version of Message #4 did XX receive</u>?

[*YY's draft / Alternative*]

Could you indicate the concrete elements (words, sentences, expressions etc…) on which your answer is based?

SECTION 5 – ***Case structure and communication critical points***

Focusing on the communication aspects of our case, we can synthesize its structure as in Table S1, which accounts also for the critical points of the interaction between the employee and the architect. Such scheme can be translated in plain language as it follows: apparently, the employee (working for the architect’s same corporation but belonging to a different branch, with no executive commission) was complaining, through Message #1, about the quality of the heating plant installation. However, some lacks of matter (for example the claimed “flaws” were not specified) suggest to figure out possible different reasons.

The architect’s first answer (Message #2) can be interpreted as an attempt to quickly end the interaction; however, the reaction of the employee (Message #3) demonstrates the failure of this tactic. It is particularly worth quoting a possible threat contained in that message, considering that XX literally writes: “if the work had regarded my home… there’s a matter of public money…”. She was hinting to the fact that the Project funding involved public sources. All this should arouse alarm and caution.

On the contrary, the architect’s spontaneous reaction (Message #4, “Hard” version, in short Msg #4/H) follows the escalation initiated by the employee: he squabbles, with a repeated retaliation, about the question of money; he expresses doubts about the fondness of the employee’s statements (“once the real existence of the problems you have marked will be assessed, I will certainly solve them…”); he substantially refuses to establish any relationship with the employee, putting just a hint of appreciation at the end of the message (“This said, I have found your objections very

interesting…”), at the same time counterbalancing it with his doubts. The most probable result should be an escalation of the conflict.

Now, if we analyse in deep Msg #4/H (the “Hard” version of Msg #4) structure, we can detect in it five main content blocks (they are marked through specific sub-headings along the message text). Msg #4/S (the “Softer” version, suggested to YY by one colleague of his) maintains the same content while its written form is reviewed and its sequence modified. In practice, the “alternative message” #4/S presents the same content blocks of Msg #4/H in a different order and under a new written form. We have synthesized a comparison of the two structures in Table S2.

The substantial difference between the “Hard” and the “Softer” versions of Message #4 is founded on the diverse approach to the arising conflict: while the “Hard” spontaneous reaction of YY approached it through a direct confrontation, the alternative “Softer” version maintains the same information content but approaches the relation with XX in terms of welcome and acknowledgement.

PART II - The collected data

SECTION 6 – *About the sample*

Our work was aimed to explore the process of message interpretation, sharing the general assumption that the communication process is universally uniform. We mean that human communication, although its expressions appear extremely variable, must however stem from a unique base of fundamental factors and processes. Something like a limb in a heterogeneous sample of humans: its aspect looks very different in function of sex, age, size, health and so on; nonetheless, it is based on a unique anatomical and functional scheme. For this, the sample's representativeness with respect to the Italian people was not critical. Thus, we decided to increase, as much as possible, the amount of participants while easing the sampling process (see research protocol, in this Supporting Information, Section 3, point 12).

We recruited 102 participants in our sample, whose characteristics are displayed in manuscript Tables 1-3. The total sample composition (manuscript Table 1) shows an exceeding rate of women vs. men and of Graduates/Post-graduates vs. High-school degree granted members (columns "Education", "Gr" bin vs. "Dg" bin; people granted with Elementary degree are inessential, only 4 out of 102). We also highlight the high rate of students and unemployed vs. employed members (columns "Employment", "E" and "F" bins vs. others). For these reasons, even if sample statistical analysis is less relevant in our work, we have drawn more balanced sub-samples from the total sample. The statistical distribution results, observed on the total sample, have been verified on

sub-samples every time it turned out necessary. The first sub-sample (“AGE”, manuscript Table 2) is exclusively composed by people over 29 years-old (age bins B, C and D, excluding A; in total, 60 members). The second one (“EMPLOYMENT”, manuscript Table 3) is exclusively composed by employed people (A to D bins, excluding E and F, i.e. students and unemployed people; in total, 65 members).

SECTION 7 – *The harvest*

In this section we present in detail an assessment about the amount of the collected materials ("how much" the respondents have written in their answers, the "physical amount" of the answers).

Starting data analysis, we firstly transcribed into a .xls file the filled questionnaires; such file turned into 1 tab containing 8 data-sheets, one for each main question or data source (information for disaggregating data, Questions #1-a, #1-b, #1-c, #2, #3, #4, Final question). Secondly, we reviewed transcriptions with regard to text correction (typos) and we harmonized data entries (different operators had produced little differences in managing spaces near punctuation marks and in using suspension points, abbreviations and similar details). At this point, it was possible to measure the collected data amount:

- Paper archive: each participant provided a 6 pages long document. Four pages contained the information materials (the title page and the transcriptions of the messages). In a few cases, on those pages, respondents had written very short notes and underlined some words. The other two pages contained the answers, which are the actual data source of our research. In conclusion, we collected 102 x 2 = 204 handwritten pages containing data to be processed.
- Digital archives: they contain the transcriptions of opened answers (harmonized text), that returned totals of 16,094 words, corresponding to 89,685 characters (spaces excluded) or 104,200 characters (spaces included).

- In order to let the readers estimate the amounts better, we calculated that using Times New Roman font, 12 size characters, space 1, with a "letter" page format and 1'' for all margins, the opened answer texts should be occupying about 26.7 to 27.4 pages (range of 3,800-3,900 characters per page, spaces included, text only, no picture, table or main titles).
- We also calculated the filling rate of the questionnaires (opened answers) in the following way: we excluded the two opened items of Question #2 (answering the opened part of the question was under condition and it was performed by just 60% of the sample); then, we recorded 27 unanswered items on an expected total of 102 participants x 11 items = 1,122 answers (see SI, Section 4, questionnaire summarizing form). The filling rate is: (1,122-27)/1,122x100 = 97.6%.
- This last information says which percentage of the opened questions received an answer but says nothing about the length of those answers. We can calculate an average length in two ways: the first is dividing the total words by the amount of participants and, then, by the amount of the opened items. The result is 16,094/102/13=12.1 words per respondent per item (answers to Question #2 are included in this calculation). In order to appreciate this value better we can follow the second way: one page, of the previously approximated 27, has typically 44 lines, which means an average of about 1 typed line per respondent per item (44x27/13/102=0.90 typed lines, answers to Question #2 included). 1 typed line is up to 90 characters (spaces included) or about 10 to 15

words; a satisfactory result, about the accomplishment of their commission by the sample members.

- About the closed answers, only the Final question is relevant (for the closed part of Question #2, see previous points), and 101 out of 102 answered to it.

In the end: survey returned a good harvest, consistent with our expectations and with the research needs.

SECTION 8 – *Data quality check: compliance with research requirements and technical-theoretical questions related to answer interpretation*

a – <u>*Answer general features and compliance with research requirements*</u>. A first noticeable aspect is that it is not possible, in any of the answers, to find overt doubts, uncertainty statements, declarations of impossibility to answer, indications of equivalent alternatives[3]. For each respondent, his/her own interpretation seems to be **the only available option**. This happens in spite of the fact that about 28% of the total sample describes the effects of the "Hard" and of the "Softer" versions of Msg #4 as similar: for an 18% (18 people) they both will solve or ease the contrast; for a 10% (10 people) they both will escalate the contrast (see this SI Section 11 and Table S5, “Total sample” columns, H+/S+ and H-/S- cells). This observation confirms that the answers are spontaneous and that our survey collected subjective perceptions, instead of elaborated rational reflections. That is what we aimed to, while following the research guide-lines and protocol (see this SI, Sections 1 and 3)[4].

Another important point is that no one of the sample members uses any technical word or expression. About this, it is worth considering how participants reacted to the two points which, from a communication slant, can be rated as the most critical: the possible threat XX expressed in Message #3; the squabbling and the personal attack by

[3] Just 1 participant (out of 102) declares some uncertainties in his final choice, writing that the final effect (as it appears in Message #5) could be obtained both with the “Hard” or the “Softer” version of Message #4. Nevertheless, while answering to the other questions, his statements are in all similar to the other participants’ ones.

[4] Exactly in order to facilitate such result, in the actual survey sessions (lasting range: 20 to 45 minutes) no discussion about the answers was allowed before the filled in questionnaires had been collected by the conductor; in addition, no further contact with the questionnaires was permitted after the sessions were over.

YY against XX in Message #4/H (see also this SI Section 5 and Table S1). Even if some participants refer to these passages in their answers, none stresses them as particularly critical and almost none labels them as “threat” or “personal attack”. Finally, while examining the answers to Questions #3 and #4 and to the Final Question, we found that about one fourth of the sample (mean for the three questions 26.5%, range 16% - 36%) overtly stated, at least once, the impossibility to analytically answer to the second part of the questions (which requested to point out the “concrete elements” that induced the answer to the first part). These respondents describe their answers to the first part of the questions as the result of “a general impression”, “a sensation/a perception”; in other cases they present such answers as “an opinion drawn from the whole message” or something similar. These observations confirm the general naïve condition of the sample about human communication (another feature requested by our research plan).

b – *About the questionnaire interpretation*. Interpretation problems, related to the questionnaires, are essentially of two kinds: interpretation of the questionnaire questions by the sample; interpretation of the sample answers by the research team. Following here, two selected examples of the first kind:

1. Question #1 (“What do you think is going on, between XX and YY?”) – It has been interpreted, in certain cases, in terms of interpersonal relationship, in other cases in terms of organizational position or professional profile.
2. Questions #1 and #2, first part (each containing indications for focusing on a specific message, out of the first three) – Actually, a large part of the sample

answered ignoring indications and simultaneously referring to all the three messages.

Here, two examples of the second kind:

3. Question #1 (“What do you think is going on, between XX and YY?”) – In one of the answers, Message #2 is defined as “bureaucratic”; however, it is impossible to understand if this adjective is used with a technical meaning (referring to a normal interaction inside an office) or with a relational one (defining a conflict, with YY using formality to resist to XX’s action). We found other similar cases.
4. Question #2, first part (requesting if, after comparing Message #3 with Message #1, the respondent considers XX’s position as “changed”) – It is interesting to see that 41 people (40% of the sample) answered “NO – Not changed”, and 61 (60%) answered “YES – It has changed”. These answers are nonetheless unsuitable for deep quantitative analysis because of the different interpretation of the word “changed”. For example the answer “YES” (the position has changed) may correspond to the actual perception of an escalated interaction; however, it may also be simply connected with attention on isolated linguistic elements (like some technical terms, introduced in Message #3 but absent in #1). The answer “NO” (no change detected) could mean that the respondent does not actually perceive any difference; it may also indicate that the differences, clearly detected relationship-wise, are nevertheless

considered scarcely effective on the respective organizational positions of XX and YY.

As stated in the research protocol (previous Section 3, point 8.), given the impossibility of a completely unambiguous formulation of concepts in natural language, we ex-post discarded from quantitative analyses all the unsuitable data.

SECTION 9 – ***Data quality check: analysis of the collected data distribution***

In order to check the existence of possible imbalances in the collected data, we explored the distribution of the answer texts with respect, by one hand, to the questionnaire questions/sub-questions and, by the other hand, to the respondents. We quantified these texts through the amount of words and characters contained in the filled questionnaires. We remind that each question/sub-question was divided into two items; when we refer to "totals", we mean that the presented data are the result of summing values related to the "strict" answer (first item, i.e. first part of the question) and values related to the indicated "concrete elements" (second item, i.e. second part of the question).

a – *Text amount distribution with respect to items*. The results of this first analysis are displayed in Table S3 and Fig. S1. Table S3 shows totals and some statistical indexes with regards to the distribution of the answer text amounts on questions/sub-questions. Data referred to all the answers (left part) are compared with those excluding Question #2 (right part). The reason of such exclusion: answering was under condition and Question #2 was answered by only a part of the sample. In order to investigate the distribution shape, we drew the histogram of Fig. S1, which displays the percent distribution of text amounts (in terms of words and characters, Question #2 excluded) with respect to the questionnaire items. It shows evident lower levels for Questions #1-b and #1-c (whose minimum, all the same, is around 7%); the rest of the values seesaws between 9% and 11% (the general percent mean, per item, is 100:11=9.1%, see Table S3, right part, "% Gen. means per item" row).

About this, we must consider that several respondents answered in short to sub-questions #1-b and #1-c, just indicating some references to the previous sub-question (#1-a, indeed having the highest values). Thus we prefer to use, for comparing different items, values referring to the percent mean of the three sub-questions of Question #1, that is 8.3% both for words and for characters (SI = spaces included). On the whole, we have a range oscillating between 8.3% and 11.1% (for words) or 11.3% (for characters). No meaningful difference is recordable; the distribution of the answer texts with respect to the questionnaire items has an almost rectangular shape and can be assessed as satisfactorily uniform. Actually, no question at all has been neglected by respondents.

b – *Sample distribution with respect to the text amounts*. The results of this analysis are displayed in Table S4 and Fig. S2 and S3. Table S4 shows totals and some statistical indexes referred to the amounts of text (in terms of words and characters, Question #2 excluded) provided by respondents through their answers. Data are displayed separating values referred to the first item of the questions ("strict" answer) from those referred to the second one ("concrete elements"). In order to investigate the distribution shape, we drew two histograms, in which participants have been grouped in bins referred to words (30-word bins, Fig. S2) and characters (200-character bins, Fig. S3, SI=spaces included) amounts. The histogram shapes have features comparable to a bell-curve, even though imperfect (see statistical details in the figure captions). Data uphold the idea of differences mainly due to spontaneous random variations and lead to the conclusion that also such distribution can be considered thoroughly acceptable (no participants seem to have neglected their commission).

PART III - Added materials

SECTION 10 – ***Analysis of some indicated component distributions***

In this Section we detail the results of our check on the distribution of the components indicated by participants, searching for possible imbalances that could contradict our findings presented in the main text (about components, see manuscript, Section “Results/1”, Sub-section *Answers to the second input of the questions)*.

Firstly, we have checked the percent distribution of components under two profiles: (i) With respect to questions/sub-questions of the questionnaire (Fig. S4, which presents an almost rectangular shape, showing that no question has been privileged or neglected in the participants' indications) ; (ii) With respect to component types (Fig. S5, types approximately ranked by physical dimension, histogram presenting an almost “bell-curve” shape, hinting to a spontaneous variability of the sample).

Secondly, we have checked the percent distribution of the sample under two profiles: with respect to the amount of component types employed (Fig. S6) and with respect to the amount of components indicated (Fig. S7). Both the histograms show “bell curve” shapes, which we assumed due to the spontaneous variability of the sample, without special imbalances to be considered.

SECTION 11 – ***Complement materials on coherence investigation***

In this Section we present some complement data, and the related analysis, referred to the investigation of the coherence among participants' interpretations and choices.

a – *From the interpretation of Message #4 two versions to the coherence assessment*. In this sub-section we report some complement data regarding the passage from the interpretations of the two Msg #4 versions (the “Hard”/H and the “Softer”/S) to the conceiving and using of the “coherence” statistical indicator (coherence between the interpretations provided by participants and the final choice they expressed).

The interpretations of the “Hard” and “Softer” versions are expressed in terms of predictions about their effects on XX through the dummy variable “Expected effects”, which can take the values “+” (if the interpretation entails the prediction of solving or easing the conflict) or “-” (if the interpretation entails the prediction of a surge or escalation of the conflict). Table S5 displays data in a dichotomous representation and shows that there is a clear convergence on the “H-/S+” combined prediction (further details in manuscript, Section “Results/2”, Sub-section *The coherence between interpretation and choice*).

Table S6 displays the participants' combined predictions cross-checked with the final choice (i.e. the answer to the Final Question, asking which of the two versions could elicit the final XX's reaction, reported by Message #5). The most frequent combined prediction (H-/S+) appears to be strongly associated to the “Softer” message choice

(further details in manuscript, Section "Results/2", *Sub-section The coherence between interpretation and choice*).

Table S7 displays the scale of the coherence indicator, i.e. the levels through which the coherence between, on the one hand, the interpretations of the "Hard" (H) and the "Softer" (S) versions of Message #4; and, on the other hand, the final choice, is rated (see manuscript for details about the definitions of the four levels of coherence set by the authors). The use of this scale allowed the study of the sample distribution with regards to the aforementioned coherence.

b – *Graphic representations complement materials*. In this sub-section some complement materials are presented regarding some graphic representations of sample distributions with respect to the coherence between the predictions about the effects of the "Hard" (H) and the "Softer" (S) version of Msg #4 and the final choice between them.

In Fig. S8-S9-S10-S11 each histogram compares the percent distributions of "H" and "S" choosers with respect to the aforementioned coherence (expressed through the coherence indicator). Each histogram regards a specific sub-sample; the displayed sub-samples are: Male, Female, High-School degree granted, Graduated participants. All the histograms show a clear difference in the distribution shape of "H" with respect to "S" choosers (further details in manuscript, Section "Results/2", Sub-section *The coherence between interpretation and choice*).

In addition, Table S8 and Table S9 contain the source data for the manuscript Fig.7 and Fig.8, which display, for the sub-samples “Age” and “Employment”, the same kind of histograms of Fig. S8-S9-S10-S11.

SECTION 12 – ***The "block preference" analysis***

The second indicator we have used (block preference indicator), was built starting from the consideration (this SI, Sections 4 and 5) that the "Hard" (H) and the "Softer" (S) version of Message #4 contain the same content blocks (it was an overt decision of YY's "colleague"), differing for the order of presentation and for linguistic form. Each block is identified as concerning a given content (see this SI, Section 5 and Table S2). Then, we investigated about possible differences regarding the attention paid by "H" and "S" choosers to different blocks, while answering to Questions #3 and #4 (predictions of the messages' effects on XX). Our goal was to explore finer characteristics in the choice process. Specifically, we intended to verify if the different choices ("H" or "S") were linked to differences in focusing on the blocks or in detecting diverse characteristics inside same blocks. In the first case the different contents, ascribable to the different blocks, would lead the process; in the second case, other factors would play a critical role.

To build the block preference indicator we, at first, examined the answers to Questions #3 and #4 and highlighted all the direct references to Message "H" and Message "S" texts (i.e. sentences in quotation marks or undoubtedly referring to clearly identifiable passages). Then, we associated them to the text blocks. Results from this part of the analysis are displayed in Tables S10-S13[5]; they contain clear indications about the

[5] Tables S10 and S11 display data with regards to the amount of **references** to each block expressed by participants. In Table S10, totals for each block and each evaluated message (as well as general totals) can be higher than the people amount, given that each person can express more than one references. Tables S12 and S13 display data with regards to the amount of **participants** that referred to each block. In Table S12, totals for each block and each evaluated message must be inferior to the participants' amount; however, the general totals can be higher, given that each person could refer to more than one block.

message blocks which the attention of participants has fallen upon. We will base our analysis on Table S12 data; blocks are displayed along with the texts of the "Hard" (H) and the "Softer" (S) versions of Message #4; a comparison among them is presented in Table S2.

Regarding the "Hard" (H) message blocks, both "H" and "S" choosers express the same preference, as their attention is mainly attracted by Block #2 (from both the versions of Msg #4) in a similar proportion: (13+9)/(21+11), about 70%, for "H" choosers; (10+43)/(17+65), about 65%, for "S" choosers. Conversely, with regard to the "Softer" (S) version, "H" and "S" choosers split. Indeed, "H" choosers focus on Blocks #2 and #3 (converted numbers[6]) in a large majority: (6+10+7+3)/(18+14), more than 80%. "S" choosers focus on Blocks #3 and #4 in a minor but still strongly prevailing proportion: (34+3+35+0)/(95+7), a little more than 70%. The principal differences regarding Block #2 and Block #4 are the following: Block #2 is the paragraph through which YY refuses to engage XX's request and re-addresses XX to another account (ZZ) inside the organisation. Both "H" and "S" choosers give Block #2 a prevalent attention, when they read it in the "Hard" (H) message. However, when they read it in the "Softer" (S) version, we see that "H" choosers maintain their preference (with a little shift towards Block #3, containing specific information) while "S" choosers pay the minimum of

---

[6] We remind that Message "S" maintained the same content of Message "H", and that content was divided into analogous text blocks, but varying their sequence (besides their written form). For reliable comparing, it has been necessary to give each "S" block a "converted number", that is the same of the correspondent block in Message "H" (see this SI, Section 5, and Table S2, extreme right column). From now on, until express notice, all the numeric references to "S" blocks must be intended as converted numbers.

attention to it (18+4=22 references) moving towards Block #3 and #4 (34+3=37 and 35+0=35 references respectively).

Block #4 is the paragraph expressing YY's relational acceptance toward XX; in Message "H", it is placed at the end, immediately before the form of salute, and is scarcely considered by both sides (even if, as usual, in different proportions). Reading it in Message "S" (where it comes as second, immediately after the form of address), we see that "H" choosers confirm their neglecting while "S" choosers pay great attention to it. In other words, "H" choosers give constantly their preference to YY's refuting and, a little less, to information providing. "S" choosers vary their preferences according to the message and they seem to attribute importance to the relational block just in Message "S", even if it is present in Message "H", too.

What does this result mean? Data seemed to be insufficient for drawing reliable conclusions; for this reason, we returned to the answers' texts (answers to Questions #3 and #4, in particular the second item, "concrete elements") and discovered what it follows. First, the apparent convergence of "H" and "S" choosers behaviour, about their taking into account Message "H" (both choosers preferentially focused on Block #2), is not real: almost all "S" choosers rate the impact of Block #2 from Message "H" on XX-YY conflict as negative **for relational reasons**. It is notable that their answers are about an information that YY gives to XX (Dr. ZZ assuming a role of account) but they refer quite exclusively to the relational impact of the passage. In this way, choosers behave homogeneously and coherently select Message "S".

Conversely, “H” choosers clearly split: on the one hand, 12 of them (out of 24, 50%, see manuscript Table 14, left column, L and LM rows) express, on the “Hard” (H) message, the same negative rating of “S” choosers (XX-YY conflict escalation) and for the same reasons (relation aspects), too. Nevertheless, they eventually choose that same Message “H” providing various justifications for their choice. On the other hand, 12 of them (50%, see manuscript Table 14, left column, MG and G rows) rate the impact of Message “H” on XX-YY conflict as positive. Coherently, they choose that message but indicate final effects of different nature: XX should be “calmed”, because of the great quantity of information received. However, she could also be sorted out, just stopped despite her dissatisfaction. These 12 people behave as if they were thinking that information is what it matters and they pay little attention to relational aspects. Such situation reminds the differences between “H” and “S” choosers’ behaviours highlighted by coherence indicator analysis (specifically, the sample distribution with respect to coherence level).

We successively noted that a minority of “S” choosers, while evaluating the “Hard” (H) message, focused on Block #4 (the relational acceptance passage) and rated it, overwhelmingly, negative (4+15=19, see Table S12, Block #4 row, column “S” choosers/”H” evaluation). Some of them, for example, justify their evaluation interpreting that YY overtly declares that he does not trust XX, given that he says he reserves himself to check for the real existence of the problem, before intervening[7]. They

[7] We observe that, as widely discussed in the manuscript (specially in the Discussion section), the question is not linked to the information *per se*, nor it regards YY’s right to control. The question is “the fact that” YY decided to overtly declare, in a certain point of his message and under a certain form, his doubt and his intentions.

do not pay any importance to the formal relational acceptance that Block #4 contains. Moving to Message "S" evaluations, we face apparent divergent behaviours, as "H" and "S" choosers focus on different blocks; nevertheless, this appearance covers an actual continuity with what we observed about the evaluations on Message "H". For example, "S" choosers that focus on Message "S"/Block #4 (we remind this is the "converted" number, corresponding to the original #2, see Table S2) express positive rates for relational reasons; quite homogeneously, they hold this block responsible for solving the conflict and they constantly describe the effects of the "Softer" (S) message (and its Block #4 in particular) with words like "acceptance", "XX satisfaction", "reassuring", "XX will feel listened to", "acknowledgement", "appreciation". Conversely, "H" chooser behaviour, once again, is split: those who, regardless of their choice, rate "S" effects as positive (10+5=15, see manuscript Table 14, left column, L and MG rows), express their evaluations in terms which are very similar to those of "S" choosers: "satisfaction" of XX, "reassuring", "calming", "attention given" and so on. Nine of them, who deem "S" as negative (2+7=9, see manuscript Table 14, left column, LM and G rows), give the maximum of importance to XX notifying the necessity to refer to a different person (Dr. ZZ). Only in 2 or 3 cases we found generic comments about the excessively "diplomatic" form of Message "S".

All these observations summed up, our investigation through the second indicator helps us to answer the initial question: if the choice between Message "H" and Message "S" can be linked to differences in block focusing or to different characteristics detected inside same focused blocks. Indeed, even though our observations seem to be pointing to

the second option, we got the impression that such formulation could result weak and that the observed processes cannot be restrained to such dichotomy. Then, how can we explain our observations? The picture can be synthesized as it follows:

- When predicting Message "H" effects, both "H" and "S" choosers mainly focus on the same block but they are attracted by different characteristics: "H" choosers by its information content; "S" choosers by its relational impact.
- When predicting Message "S" effects, "H" and "S" choosers mainly focus on different blocks. However, their answers show that such behaviour is linked to the attraction they feel towards the same characteristics that stimulated them in the previous case: "H" choosers insist on privileging information content (and Blocks #2 and #3, that concentrate the information); "S" choosers shift towards new blocks that make evident the relational care of YY with regards to XX (Blocks #3 and #4, converted numbers, see Note 6).

One last aspect to be cleared: the second point contains, besides the specific divergence in focusing, a new example of the first case, i.e. the same focusing joined to attention paid to different characteristics. Actually, both "H and "S" choosers focus also on Block #3 (converted number) of the "Softer" (S) message, that is labelled as "Information" in Table S2. However, even though that block undoubtedly contains information, the two versions present it in different ways. Confronting the texts, we can easily verify that the "H" version bears just technical and formal contents while the "S" version pays attention to present the information as a "service" for the colleagues. Evidently, respondents jointly take such aspect into account but (as usual) they interpret

it in different ways. As a matter of fact, "H" choosers mainly highlight the **information** that "the works are not yet concluded and final checks… are about to be carefully planned"; "S" choosers mainly emphasize the **reassurance** (a purely relational aspect) that YY expressly gives to XX with his words "I assure you [that your indications] will not be ignored".

In synthesis, what we found is that, about focusing on blocks, the differences, as well as the convergence, are apparent and the attention of participants seems to be attracted by those blocks that can "resound" something they are possibly looking for, something pre-existent. What drives the focusing is not the mere information content of the blocks. Once more, we have observed nothing else than a "disassembling" operation (see manuscript for details). In doing so, we have collected two examples of what kind of "pre-existing blueprints" (in some way present in the actors' central nervous system) can orient focusing and explain the different approaches employed by "H" or "S" choosers: the first mainly focus on content or context aspects; the second ones mainly focus on relational aspects.

References

De Mauro T. 2003 (1980). *Guida all'uso delle parole*. Roma: Editori Riuniti.

SUPPORTING INFORMATION Figures

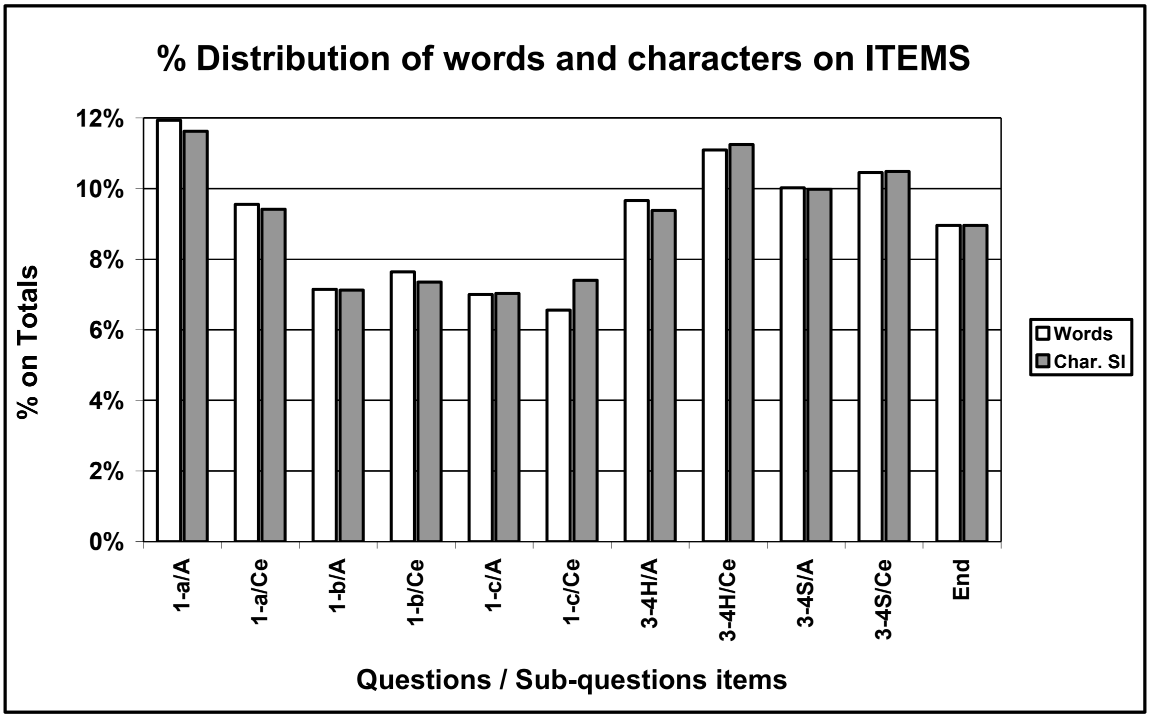

Figure S1: Percent distribution of words and characters on question items (Question #2 excluded).

[Legend: 1-a, 1-b, 1-c = Answers to sub-questions of Question #1; 3-4/H, 3-4/S = Answers to Questions #3 and #4 referred to Message “H” (the “Hard” version of Msg #4) or to Message “S” (the “Softer version); End = Final question; A = “Strict” answers; Ce = Concrete elements; Char.SI = Characters (spaces included)]

This histogram shows that the word and character percent amounts resulting from the respondents’ answers vary, with respect to items, from 6.6% to 11.9% (words) and from

7.0% to 11.6% (characters, spaces included). The range reduces to 8.3%-11.1% (words) and 8.3%-11.3% (characters SI) if the three sub-questions of Question #1 are grouped together and their mean is considered (see text for details). The amounts appear to be distributed in an almost rectangular distribution (i.e. in a satisfactorily uniform shape) across the questions of the questionnaire. On the whole, no item seems to be definitely privileged, or neglected, by participants.

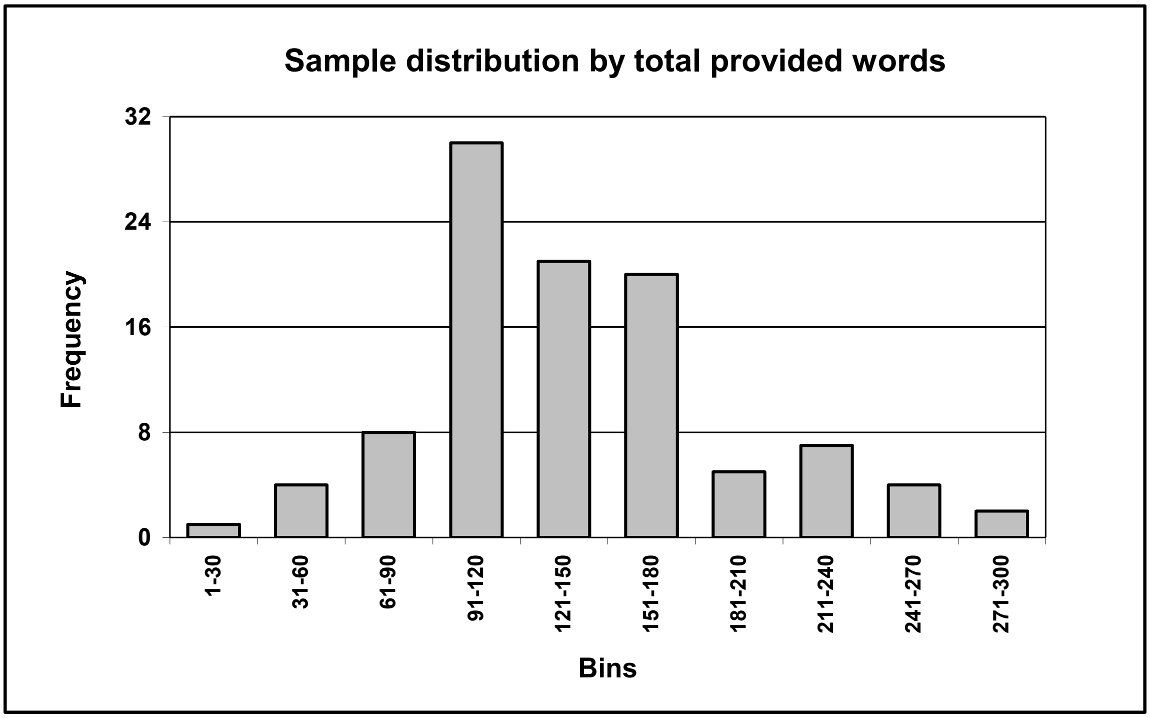

Figure S2: Sample distribution with respect to total provided words (Question #2 excluded).

The histogram shows how the sample is distributed with respect to the amount of words provided by participants. The participants are grouped in 30-word bins. Totals ("strict" answers + concrete elements indications) are displayed. The main statistical indexes of the distribution are the following (SD = Standard deviation; CV(%) = percent Coefficient of Variation):

Mean = 138.5; Median = 131; Mode = 142; SD = 53.7; CV(%) = 38.75%.

Skewness = 1.15; Kurtosis = 0.09.

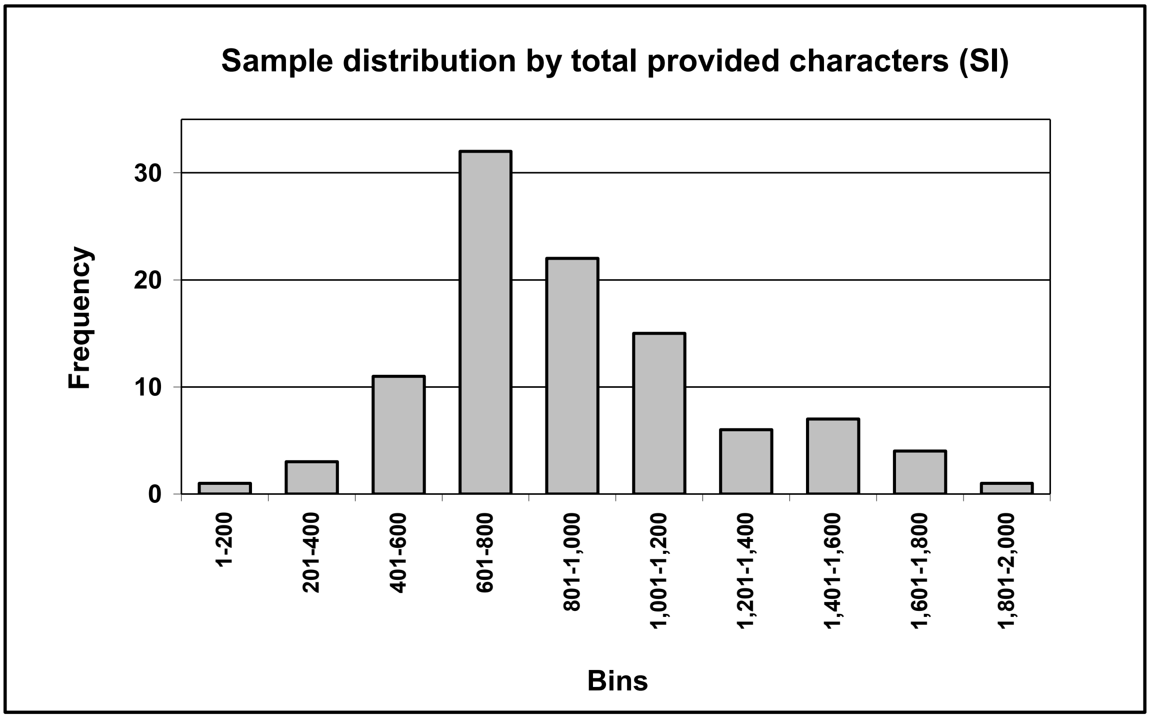

Figure S3: Sample distribution with respect to total provided characters (spaces included, Question #2 excluded).

The histogram shows how the sample is distributed with respect to the amount of characters (spaces included) provided by participants. The participants are grouped in 200-character bins. Totals ("strict" answers + concrete elements indications) are displayed. The main statistical indexes of the distribution are the following (SD = Standard deviation; CV(%) = percent Coefficient of Variation):

Mean = 900.4; Median = 813; Mode = 1,040; SD = 341.6; CV(%) = 37.94%

Skewness = 1.31; Kurtosis = 1.12.

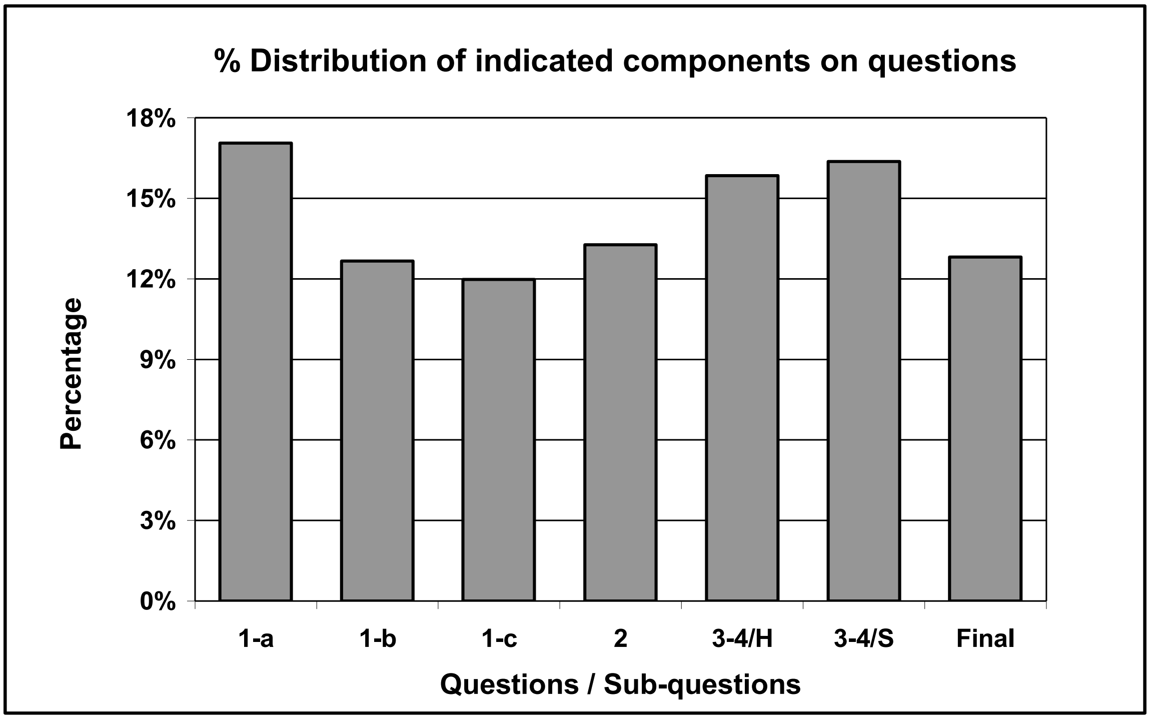

Figure S4: Percent distribution of indicated components with respect to questions/sub-questions.

With respect to questions, the respondents' indications about the focused components (see text for definition) present a rectangular-like percent distribution (differences in a range around 5%, from 12% to 17% about, source data from Table 8, "%" column). The range reduces to around 3.6% (from 12.8% to 16.4% about) if we group together the three sub-questions of Question #1 and consider their mean (the reason is that the answers to Questions #1-b and #1-c are often given in short, indicating reference to the already provided answer to Question #1-a). The indications are distributed without any significant imbalance among the different questions of the questionnaire. The approach through subjective selective focusing does not definitely privilege any question or item.

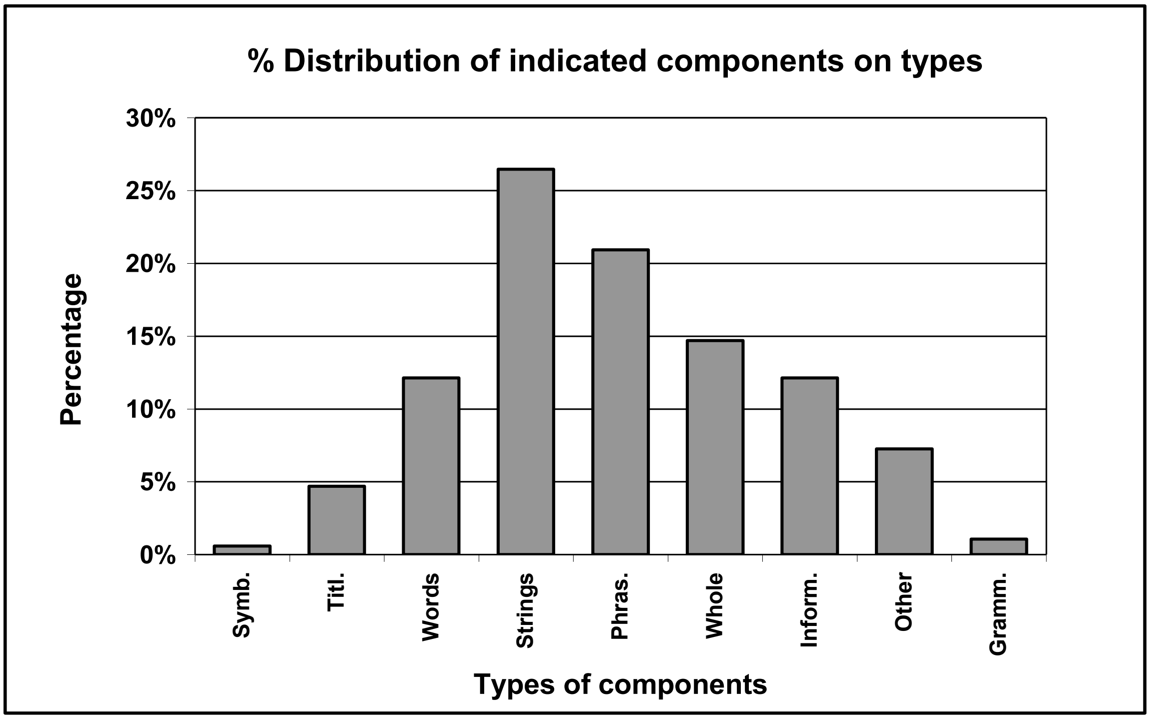

LEGEND: Symb. = Punctuation marks; Titl. = Title/salutes (opening and closing expressions); Phras. = Complete phrases/periods; Whole = References to the message as a whole; Inform. = Information content; Gramm. = Grammar notations (verb tense etc.)

Figure S5: Percent distribution of indicated components with respect to component types.

The respondents' indications have been grouped in bins by type. The presented percent distribution (source data from manuscript Table 8, "%" row) has been built through ranking the first six types (from "Symbols" to "Whole") by their increasing size. The remaining three types (Information content, Other components and Grammar notations) have been added ranking them by decreasing percent values. The highest frequencies correspond to middle-sized "chunks" of the messages.

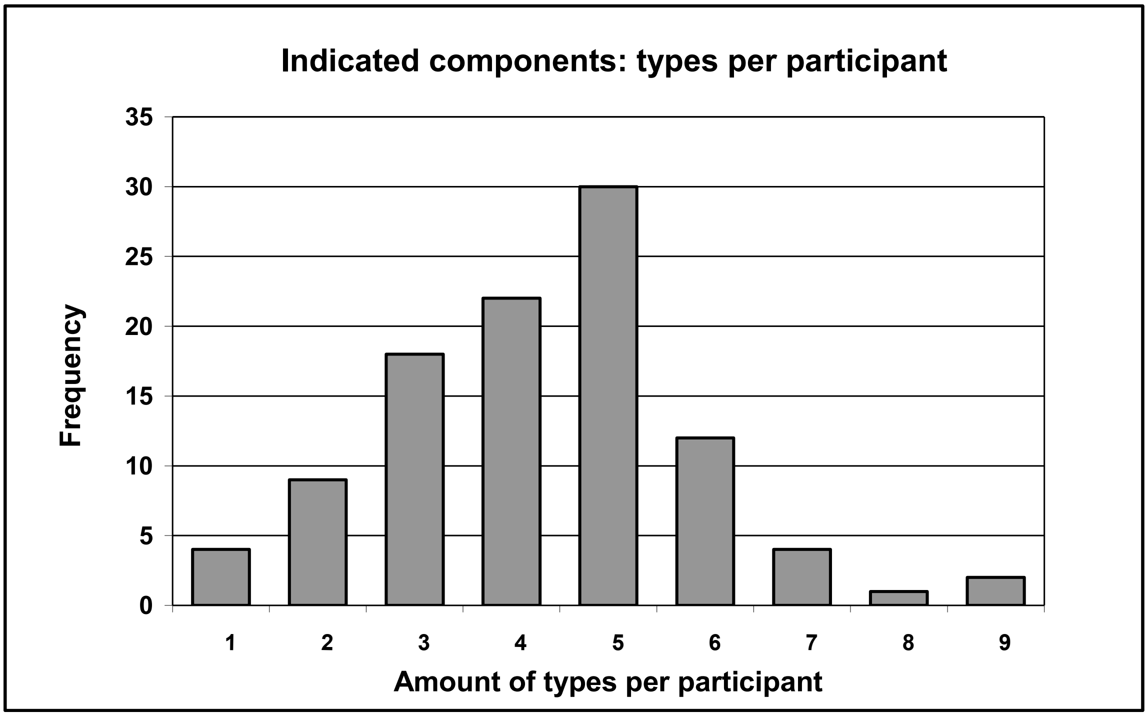

Figure S6: Sample distribution with respect to the amount of component types used by participants.

Respondents have been grouped in bins by the amount of component types (see text for definition) they used. The histogram shows the sample's distribution; it presents the highest frequencies on the 3-4-5 types-per-participant bins and has an almost "bell curve" shape. The main statistical indexes of the distribution are the following:

Mean = 4.3; SD = 1.6; Skewness = 0.25; Kurtosis = 0,49.

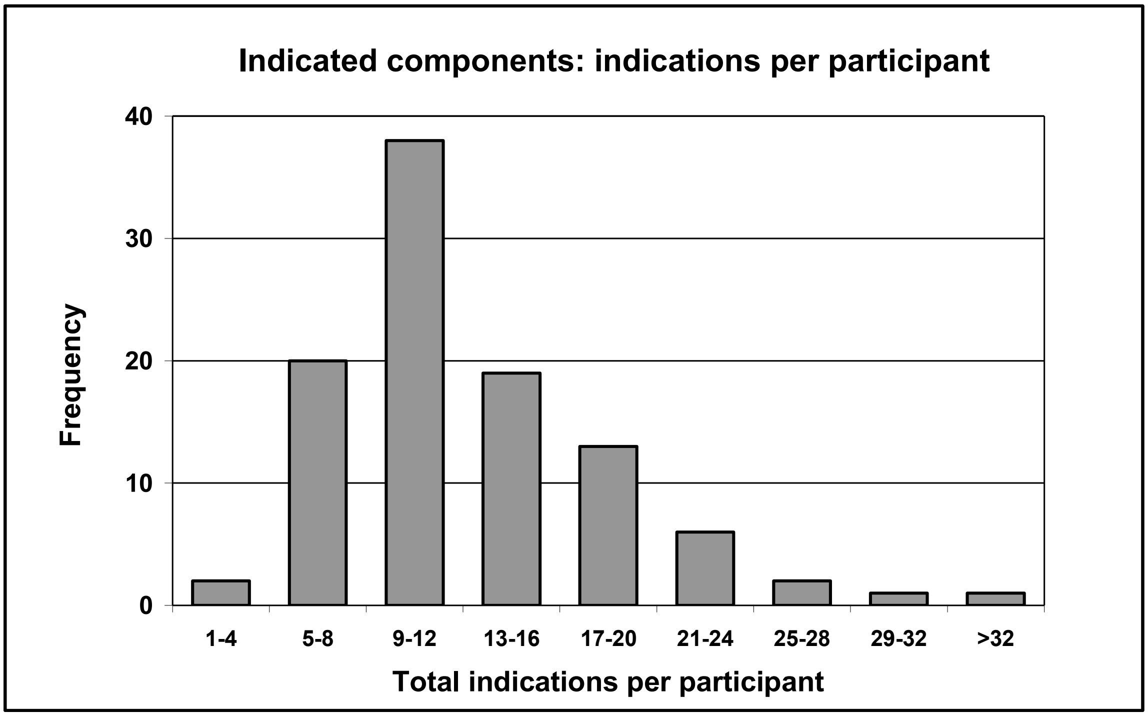

Figure S7: Sample distribution with respect to the amount of components indicated by participants.

Respondents have been grouped in bins by the amount of components (see text for definition) they indicated. The histogram shows the sample distribution; it presents the highest frequencies on the second, third and fourth bins and has an almost “bell curve” shape (even though it is clearly skewed towards the left side). The main statistical indexes of the distribution are the following:

Mean = 12.9; SD = 6.2; Skewness = 1.93; Kurtosis = 7.18.

..

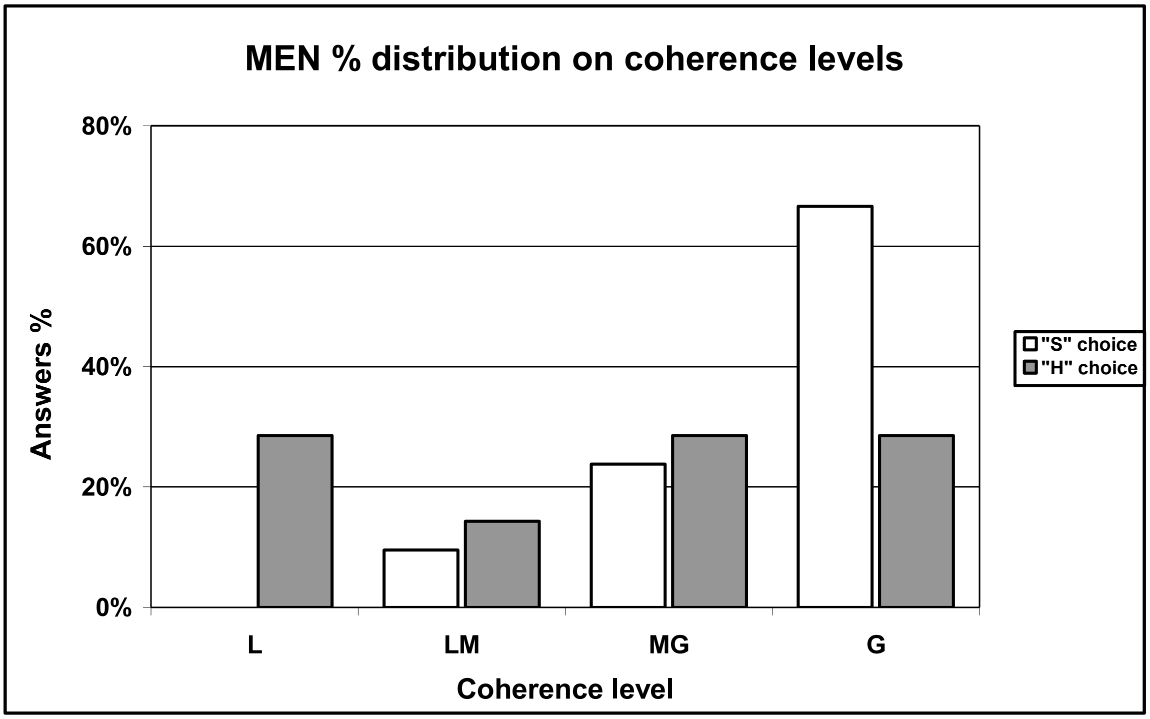

LEGEND: L = Low; LM = Low-Medium; MG = Medium-Great; G = Great level of coherence.

Figure S8: Sample percent distribution with respect to coherence levels / Comparing "Hard" and "Softer" Msg #4 version choosers / Sub-sample MEN

This histogram shows the percent distributions of MALE respondents according to the coherence (expressed through the coherence indicator) between, on the one hand, their interpretations of the "Hard" (H) and the "Softer" (S) version of Msg #4; on the other hand, their final choice between the two versions. Data is shown separately for "H" and "S" choosers. Chi-squared test unsuitable for the presence of a zero value; Fisher's Exact test: p=0.003.

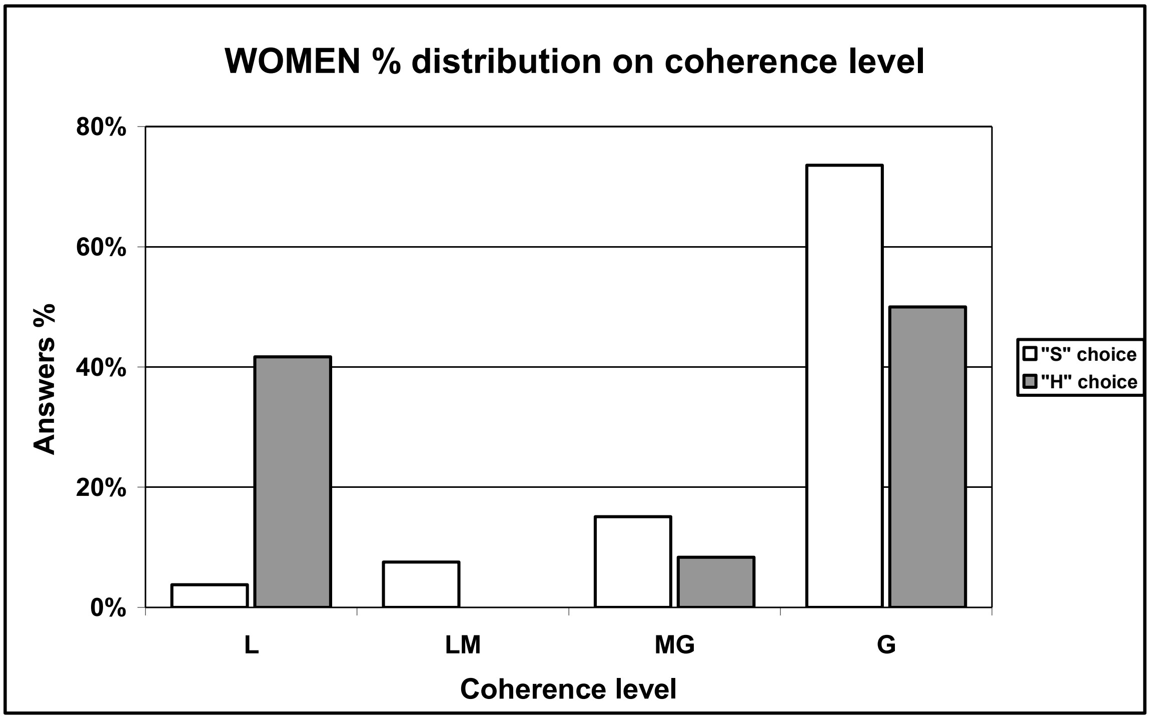

LEGEND: L = Low; LM = Low-Medium; MG = Medium-Great; G = Great level of coherence.

Figure S9: Sample percent distribution with respect to coherence levels / Comparing "Hard" and "Softer" Msg #4 version choosers / Sub-sample WOMEN

This histogram shows the percent distributions of FEMALE respondents according to the coherence (expressed through the coherence indicator) between, on the one hand, their interpretations of the "Hard" (H) and the "Softer" (S) version of Msg #4; on the other hand, their final choice between the two versions. Data is shown separately for "H" and "S" choosers. Chi-squared test unsuitable for the presence of a zero value; Fisher's Exact test: p=0.004.

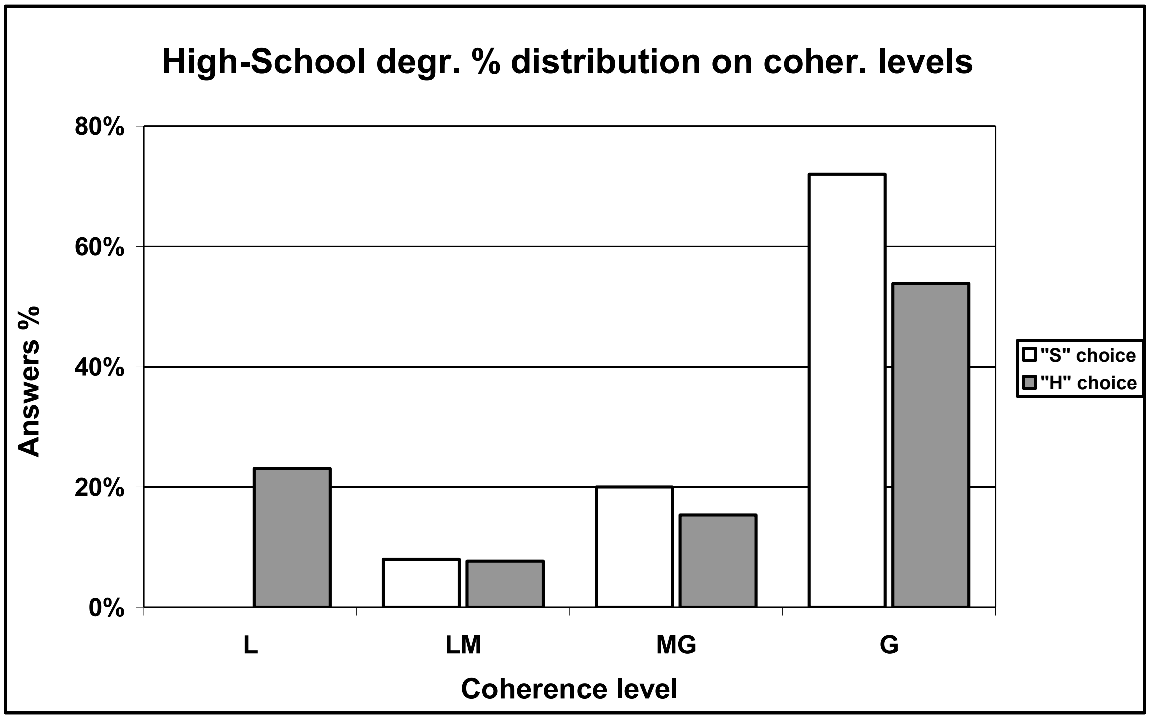

LEGEND: L = Low; LM = Low-Medium; MG = Medium-Great; G = Great level of coherence.

Figure S10: Sample percent distribution with respect to coherence levels / Comparing “Hard” and ”Softer” Msg #4 version choosers / Sub-sample High School

This histogram shows the percent distributions of HIGH-SCHOOL degree granted respondents according to the coherence (expressed through the coherence indicator) between, on the one hand, their interpretations of the “Hard” (H) and the “Softer” (S) version of Msg #4; on the other hand, their final choice between the two versions. Data is shown separately for “H” and “S” choosers. Chi-squared test unsuitable for the presence of a zero value; Fisher's Exact test: p=0.001.

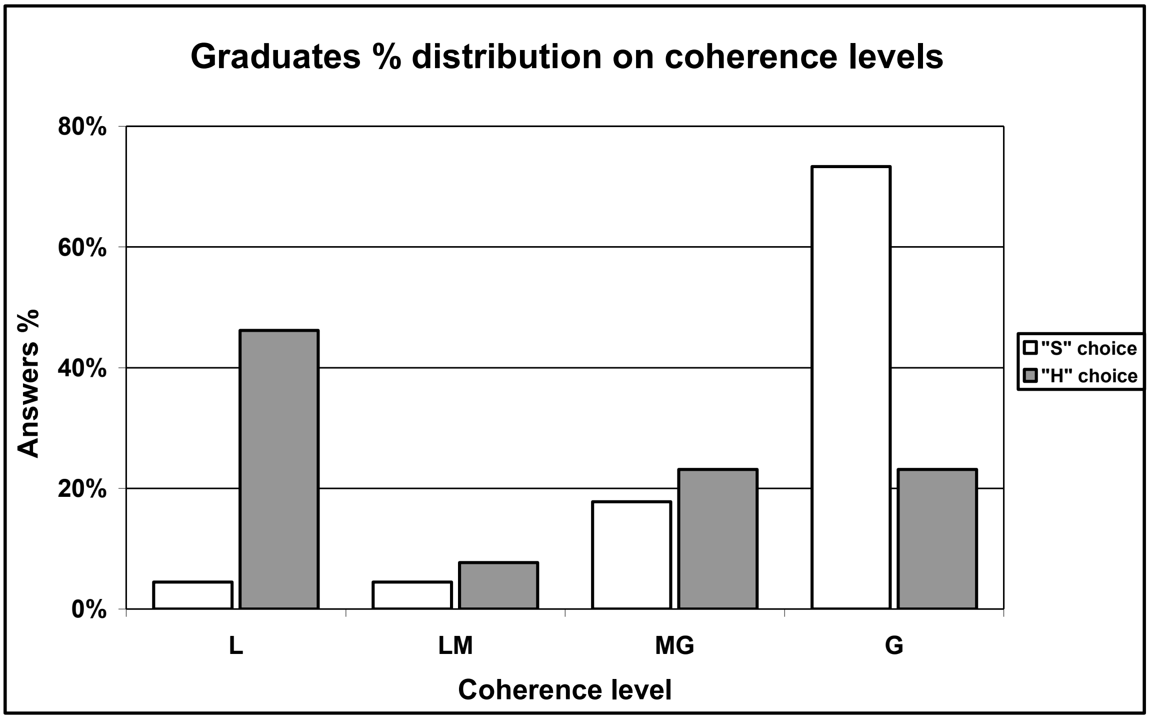

LEGEND: L = Low; LM = Low-Medium; MG = Medium-Great; G = Great level of coherence.

Figure S11: Sample percent distribution with respect to coherence levels / Comparing “Hard” and ”Softer” Msg #4 version choosers / Sub-sample Graduates

This histogram shows the percent distribution of GRADUATED respondents according to the coherence (expressed through the coherence indicator) between, on the one hand, their interpretations of the “Hard” (H) and the “Softer” (S) version of Msg #4; on the other hand, their final choice between the two versions. Data is shown separately for “H” and “S” choosers. Distributions result significantly different (Chi-squared test and Fisher's Exact test: *p*=0.001).

SUPPORTING INFORMATION Tables

Message	Author	Character	Critical points	Notes
#1	XX	The employee, woman, line position	Lack of matter: no specific claim, no evident goal (consequent suspect of relational problems).	Start message
#2	YY	The professional, man, executive in charge of the Project	Evasive action, bureaucratic answer.	First feed-back
#3	XX	The employee	Hardened position, presence of a possible threat (*ALARM*!!).	Reaction / Reinforce
#4 "H"	YY	The professional	Squabble + Refusing relational level + Personal attack to XX (*ALARM*!!).	Second feed-back

Table S1: The case structure and the communication critical points.

This scheme displays the interaction structure and the communication critical points related to the first part of the case. It considers the exchanged messages (Messages #1 to #3) and provides comments on the "Hard" version of Message #4 (spontaneously prepared by the "architect", i.e. YY). While creating our case, we figured that exactly this could be the analysis of YY's colleague (or some external communication expert) that drove him/her to suggest the alternative.

**Blocks**	**"H" Structure**	**"S" Structure**	**Conversion**
#1	Form of address	Form of address	S "1" → S "1" *converted*
#2	Re-addressing XX	Relational acceptance	S "2" → S "4" *converted*
#3	Information	Re-addressing XX	S "3" → S "2" *converted*
#4	Relational acceptance	Information	S "4" → S "3" *converted*
#5	Form of saluting	Form of saluting	S "5" → S "5" *converted*

Table S2: Comparing text blocks in the two versions (the "Hard" and the "Softer" one) of Message #4.

The message presented as alternative to Message #4/H (i.e. the "Softer" version of Message #4, in short Msg #4/S) has the same text blocks of the "Hard" version with the same information content. Only the position in the text and the written form were modified. Extreme right column shows the "conversion table" of the blocks numbers for the two versions, in order to simplify referencing while comparing them.

	All the Questions (13 items)			*Quest. #2 excluded (11 items)*		
	**Words**	**Char.(SE)**	**Char.(SI)**	**Words**	**Char.(SE)**	**Char.(SI)**
TOTALS	16,094	89,685	104,200	14,128	79,097	91,843
General means per item	1,238	6,899	8,015	1,284	7,191	8,349
% Gen. means per item	*7.7%*	*7.7%*	*7.7%*	*9,1%*	*9,1%*	*9,1%*
CV(%)	*21.0%*	*20.3%*	*20.5%*	*18.78%*	*17.19%*	*17.56%*
General means per person	158	879	1,022	139	776	900
Gen. means per person-item	12.1	68	79	12.6	71	82

Table S3: Descriptive analysis of the text amount distribution with respect to the questionnaire items.

[Legend: Char.(SE) / (SI) = Character amounts, (Spaces Excluded) / (Spaces Included); CV(%) = percent Coefficient of Variation]

The table shows totals and some statistical indexes (some means and percent coefficient of variation) referred to the word and character amounts resulting from the texts of the respondents' answers. Indexes are calculated on question items, in two ways: on all the opened items (13 items, left part of the table); on all the items excluding Question #2 (11 items, right part of the table, see text for the reasons of such exclusion). Further information in Fig. S1.

	"Strict" answers			*Concrete elements*			*Totals*		
	**Words**	**Ch.(SE)**	**Ch.(SI)**	**Words**	**Ch.(SE)**	**Ch.(SI)**	**Words**	**Ch.(SE)**	**Ch.(SI)**
TOTALS	6,463	35,484	41,461	7,665	43,613	50,382	14,128	79,097	91,843
% on General total	*45.7%*	*44.9%*	*45.1%*	*54.3%*	*55.1%*	*54.9%*	*100 %*	*100 %*	*100 %*
Gen. means p. person	63.4	348	407	75.1	428	494	138,5	775	900
CV(%)	*48.58%*	*43.63%*	*44.80%*	*45.56%*	*45.46%*	*45.75%*	*47.77%*	*46.13%*	*46.61%*
Minimum	8	73	76	4	25	28	4	25	28
Maximum	175	905	1,075	185	1,030	1,180	185	1,030	1,180

Table S4: Descriptive analysis of the sample distribution with respect to the provided text amounts.

[Legend: Ch.(SE) / (SI) = Character amounts, (Spaces Excluded) / (Spaces Included); CV(%) = percent Coefficient of Variation]

The table shows totals and some statistical indexes (some means, percent coefficient of variation and minimum/maximum) referred to the word and character amounts provided by the respondents through their answers. Answers to Question #2 have been excluded (see text for the reasons of such exclusion). In the left part, data from the answers to the first item of the questions ("strict" answer); in the central part, to the second item (concrete elements). Total values are displayed in the right part of the table. Further information in Fig. S2, S3.

	**Total sample**						**Sub-sample "AGE"**						**Sub-sample "EMPLOYMENT"**					
	**S+**		**S-**		***Totals***		**S+**		**S-**		***Totals***		**S+**		**S-**		***Totals***	
**H+**	18	*22.5%*	9	*47.4%*	27	*27.3%*	8	*17.4%*	4	*36.4%*	12	*21.1%*	9	*18.4%*	6	*46.2%*	15	*24.2%*
**H-**	62	*77.5%*	10	*52.6%*	72	*72.7%*	38	*82.6%*	7	*63.6%*	45	*78.9%*	40	*81.6%*	7	*53.8%*	47	*75.8%*
Tot.	***80***	*100.0 %*	***19***	*100.0 %*	***99***	*100.0%*	***46***	*100.0%*	***11***	*100.0%*	***57***	*100.0%*	***49***	*100.0%*	***13***	*100.0%*	***62***	*100.0%*
Gen. Total	***99***						***57***						***62***					

Table S5: Distribution of predictions about the effects of the “Hard” (H) and the “Softer” (S) version of Message #4 on the receiver.

Predictions about the effects that the “Hard” (H) or the “Softer” (S) version of Message #4 could have on the receiver (the “employee” XX) are independently expressed, by each member of the sample, through answering to Questions #3 and #4. Answers are classified through the dummy variable “Expected effects” (possible values “+”, if respondents point out that the message will solve the XX-YY conflict, or “-“ in the opposite case). The table shows, for the total sample and the two control sub-samples, that all the possible combinations of predictions are present. Distribution is clearly imbalanced (definite preference on “H-/S+” combination). Significance is checked through Chi-squared test (p=0.029, total sample; p=0.166, sub-sample “AGE”; p=0.038, sub-sample “EMPLOYMENT”) and Fisher's Exact test (p=0.043, total sample; p=0.219, sub-sample “AGE”; p=0.064, sub-sample “EMPLOYMENT”).

	**Total sample**						**Sub-sample "AGE"**						**Sub-sample "EMPLOYMENT"**					
	**"H" Choice**		**"S" Choice**		**Totals**		**"H" Choice**		**"S" Choi-ce**		**Totals**		**"H" Choice**		**"S" Choice**		**Totals**	
**H+/S+**	5	*20.8%*	13	*17.6%*	18	*18.4%*	4	*26.7 %*	4	*9.8%*	8	*14.3%*	4	*22.2%*	5	*11.6%*	9	*14.8%*
**H+/S-**	7	*29.2%*	2	*2.7%*	9	*9.2%*	3	*20.0 %*	1	*2.4%*	4	*7.1%*	5	*27.8%*	1	*2.3%*	6	*9.8%*
**H-/S+**	10	*41.7%*	52	*70.3%*	62	*63.3%*	7	*46.7 %*	31	*75.6%*	38	*67.9%*	8	*44.4%*	32	*74.5%*	40	*65.6%*
**H-/S-**	2	*8.3%*	7	*9.4%*	9	*9.2%*	1	*6.7%*	5	*12.2%*	6	*10.7%*	1	*5.6%*	5	*11.6%*	6	*9.8%*
Totals	***24***	*100.0%*	***74***	*100.0%*	***98***	*100.0%*	***15***	*100.0 %*	***41***	*100.0%*	***56***	*100.0%*	***18***	*100.0%*	***43***	*100.0%*	***61***	*100,0%*
Gen. Total	***98***						***56***						***61***					

Table S6: Combined predictions cross-checked with the final choice between the "Hard" (H) and the "Softer" (S) version of Message #4.

In this table the combined predictions (see Table S5) about the effects that the "Hard" (H) or the "Softer" (S) version of Message #4 could have on the receiver (the "employee" XX) are crossed with the final choices of the respondents (all the variables are independent). Data shows the association (for the total sample and the two control sub-samples) between the "H-/S+" combination and the S-version (the "Softer" one) as final choice. In addition, some correlations between the two variables is underlined by Chi-squared test (p=0.001, total sample; p=0.035, sub-sample "AGE"; p=0.009, sub-sample "EMPLOYMENT") and Fisher's Exact test (p=0.002, total sample; p=0.027, sub-sample "AGE"; p=0.008, sub-sample "EMPLOYMENT").

	**L (*low coherence*)**	**LM (*low-medium c.*)**	**MG (*med.-great c.*)**	**G (*great coherence*)**
**“H” choice**	**H- / S+**	**H- / S-**	**H+ / S+**	**H+ / S-**
**“S” choice**	**H+ / S-**	**H- / S-**	**H+ / S+**	**H- / S+**

Table S7: Plot of the coherence level scale.

The table shows the scale of the coherence levels expressed through the coherence indicator; four levels of coherence are defined and ranked. The indicator rates the degree of coherence between two independent kinds of answers each respondent provided in his/her own questionnaire. On the one hand, the answers to Questions #3 and #4, reporting the respondent's predictions about the effects that the “Hard” (H) or the “Softer” (S) version of Message #4 could have on the receiver, the “employee” XX (the predictions are represented through the dummy variable “Expected effects” and labelled “+” if they indicate that the message will ease or solve the contrast between XX and YY, “-“ in the opposite case). On the other hand, the answer to the Final question, reporting the final choice the respondent made indicating which, between the “Hard” and the “Softer” version of Message #4, was suitable to solve the case ending the XX-YY conflict.

**"H" Choosers**			**"S" Choosers**			**Total**	
Coherence level	***Values***	***%***	***Coherence level***	***Values***	***%***	***Values***	***%***
**L** (H-/S+)	7	*46.7*	**L** (H+/S-)	1	*2.4*	8	*14.3*
**LM** (H-/S-)	1	*6.7*	**LM** (H-/S-)	5	*12.2*	6	*10.7*
**MG** (H+/S+)	4	*26.7*	**MG** (H+/S+)	4	*9.8*	8	*14.3*
**G** (H+/S-)	3	*20.0*	**G** (H-/S+)	31	*75.6*	34	*60.7*
Total	**15**	***100.0***	***Total***	**41**	***100.0***	**56**	***100.0***

LEGEND: L = Low; LM = Low-medium, MG = Medium-great, G = Great level of coherence between predictions and choice; H/S = Versions of Message #4; +/- = type of predicted effect (resolution or escalation of the conflict) of the messages on XX.

Table S8: Sample distribution with respect to coherence levels and expressed choice (Sub-sample "Age").

The table displays (for the sub-sample "Age", >29yy-old people only) the distribution of participants with respect to the coherence between, on the one hand, the predictions about the effects on XX of the "Hard" (H) and the "Softer" (S) version of Msg #4; on the other hand, the final "H-or-S" choice. Data is displayed separately for "H" and "S" choosers and highlights a clear correlation between the two variables coherence and choice; Chi-squared test and Fisher's Exact test return high significance (p<0.001).

**"H" Choosers**			**"S" Choosers**			**Total**	
Coherence level	***Values***	*%*	***Coherence level***	***Values***	*%*	***Values***	*%*
**L** (H-/S+)	8	*44.4*	**L** (H+/S-)	1	*2.3*	9	*14.8*
**LM** (H-/S-)	1	*5.6*	**LM** (H-/S-)	5	*11.6*	6	*9.8*
**MG** (H+/S+)	4	*22.2*	**MG** (H+/S+)	5	*11.6*	9	*14.8*
**G** (H+/S-)	5	*27.8*	**G** (H-/S+)	32	*74.4*	37	*60.7*
Total	**18**	***100.0***	***Total***	**43**	***100.0***	**61**	***100.0***

LEGEND: L = Low; LM = Low-medium, MG = Medium-great, G = Great level of coherence between predictions and choice; H/S = Versions of Message #4; +/- = type of predicted effect (resolution or escalation of the conflict) of the messages on XX.

Table S9: Sample distribution with respect to coherence levels and expressed choice (Sub-sample "Employment").

The table displays (for the sub-sample "Employment", people with a regular employment only) the distribution of participants with respect to the coherence between, on the one hand, the predictions about the effects on XX of the "Hard" (H) and the "Softer" (S) version of Msg #4; on the other hand, the final "H-or-S" choice. Data is displayed separately for "H" and "S" choosers and highlights a clear correlation between the two variables coherence and choice; Chi-squared test and Fisher's Exact test return high significance (p<0.001).

**Blocks**	**"H" Choosers**				**"S" Choosers**			
	**"H" Evaluation**		**"S" Evaluation[*]**		**"H" Evaluation**		**"S" Evaluation[*]**	
	+	-	+	-	+	-	+	-
1	0	0	0	0	0	1	1	0
2	16	13	6	10	13	75	23	4
3	6	1	7	5	5	6	50	4
4	3	1	7	1	4	16	52	0
5	0	0	1	0	0	1	7	0
TOTAL	**25**	**15**	**21**	**16**	**22**	**99**	**133**	**8**

[*] The sequence of the blocks belonging to Message "H" is the original one (as it appears in the actual message); the sequence belonging to Message "S" is *converted* (see SI, Section 12 and Note 6, for details).

Table S10: Block preference analysis (I) – Amount of expressed REFERENCES.

[Legend: +/- = type of predicted effect (resolution or escalation of the conflict) of the "Hard" (H) and the "Softer" (S) version of Msg #4 on XX.]

The table displays the "preference" for different blocks, expressed through the amount of references to each block. Data is disaggregated for H/S choice and for type of expressed predictions (+/-) on Message "H" and Message "S" effects. Respondents, while evaluating the "H" message, seem to be mainly focused on the same block (the Block #2), regardless of their H/S choice. On the opposite, while evaluating the "S" message, they mainly focus on different blocks, depending on the choice they expressed.

General Totals		*Means*
Total references to Msg "H" blocks	**161**	***1,59*** references/participant
Total references to Msg "S" blocks	**178**	***1,76*** references/participant
Total references expressed by "H" choosers	77	***2,96*** references/participant
Total references expressed by "S" choosers	**262**	***3,49*** references/participant
General total	**339**	***3,36*** references/participant

Table S11: Block preference analysis (I) – Additional data.

The table displays some additional information about data displayed in previous Table S10. Additional data consists of total expressed references and mean values about references per participant.

Blocks	"H" Choosers				"S" Choosers			
	"H" Evaluation		"S" Evaluation(*)		"H" Evaluation		"S" Evaluation(*)	
	+	-	+	-	+	-	+	-
1	0	0	0	0	0	1	1	0
2	13	9	6	10	10	43	18	4
3	5	1	7	3	3	5	34	3
4	3	1	4	1	4	15	35	0
5	0	0	1	0	0	1	7	0
TOTAL	**21**	**11**	**18**	**14**	**17**	**65**	**95**	**7**

(*) The sequence of the blocks belonging to Message "H" is the original one (as it appears in the actual message); the sequence belonging to Message "S" is *converted* (see SI, Section 12 and Note 6, for details).

Table S12: Block preference analysis (II) – Amount of PARTICIPANTS expressing references.

[Legend: +/- = type of predicted effect (resolution or escalation of the conflict) of the "Hard" (H) and the "Softer" (S) version of Msg #4 on XX.]

The table displays the "preference" for different blocks, expressed through the amount of participants that refer to each block. Data is disaggregated for H/S choice and for type of expressed predictions (+/-) on Message "H" and Message "S" effects. Respondents, while evaluating the "H" message, seem to be mainly focused on the same block (the Block #2), regardless of their H/S choice. On the opposite, while evaluating the "S" message, they mainly focus on different blocks, depending on the choice they expressed.

**General Totals**		***Means***
Total people referring to msg "H" blocks	**114**	***1,13*** referred blocks/participant
Total people referring to msg "S" blocks	**134**	***1,33*** referred blocks/participant
Total "H" choosers' block evaluations	**64**	***2,46*** referred blocks/participant
Total "S" choosers' block evaluations	**184**	***2,45*** referred blocks/participant
General total	**248**	***2,46*** referred blocks/participant

Table S13: Block preference analysis (II) – Additional data.

The table displays some additional information about data displayed in previous Table S12. Additional data consists of total people expressing references and mean values about referred blocks per participant.
